# Mining and analysis of adverse event signals of isotretinoin based on the real-world data of FAERS database

**DOI:** 10.3389/fmed.2025.1559972

**Published:** 2025-10-01

**Authors:** Liu Yang, Hanzhang Xie, Ying Jia, Bingnan Cui, Zhanshuo Xiao

**Affiliations:** ^1^Department of Dermatology, Guanganmen Hospital, China Academy of Chinese Medical Sciences, Beijing, China; ^2^College of Traditional Chinese Medicine, Beijing University of Chinese Medicine, Beijing, China

**Keywords:** isotretinoin, pharmacovigilance, FAERS database, adverse events, signal detection, pharmacovigilance methodology, Dandy-Walker syndrome, suicidal ideation

## Abstract

**Objective:**

Isotretinoin (ISO) is an oral prescription retinoid that is well-known for its effectiveness in treating acne. This study aims to provide valuable insights into the safety of its clinical use by analyzing adverse events (AEs) associated with ISO reported in the FDA Adverse Event Reporting System (FAERS) database.

**Methods:**

FAERS data from Q1 2004 to Q2 2024 were analyzed. Duplicate reports were excluded, and disproportionality analysis using four algorithms (ROR, PRR, BCPNN, EBGM) was performed. AEs were classified using MedDRA terms, with Important Medical Events (IMEs) flagged based on the MedDRA IME list.

**Results:**

The FAERS database identified 46,526 ISO-related AE reports covering 27 System Organ Classes (SOCs). Of these, 445 AEs showed significant signals across all four algorithms, including 38 previously unlabeled AEs within the top 50 positive Preferred Term (PT) signals. Key IMEs, such as Dandy-Walker syndrome (*N* = 13), childhood depression (*N* = 3), and suicidal ideation (*N* = 2,175), were identified, with Gastrointestinal Disorders being the most frequently reported SOC (10,064 reports). AEs occurred most frequently in the early (0–30 days, 23.5%) and mid-treatment phases (91–180 days, 22.7%), with females accounting for 55.9% of the reports.

**Conclusion:**

This study identified previously unlabeled AEs and IMEs associated with ISO, including life-threatening events such as Dandy-Walker syndrome, suicidal ideation, and childhood depression, emphasizing the need for updated drug labels and enhanced safety measures. Future research should focus on mechanisms underlying teratogenicity and psychiatric risks to refine ISO’s safety profile.

## 1 Introduction

Isotretinoin (ISO), a synthetic derivative of vitamin A (13-cis-retinoic acid), has been a cornerstone therapy for severe nodulocystic and papulopustular acne since its approval by the U.S. Food and Drug Administration (FDA) in 1982 (1–3). Its mechanism of action—suppressing keratinocyte hyperproliferation, reducing sebum production, and inhibiting *Propionibacterium acnes* growth—results in significant improvement in acne symptoms (4, 5). Beyond acne, ISO is also effective in treating related conditions such as rosacea, gram-negative folliculitis, and hidradenitis suppurativa (6, 7). As the only medication with long-term efficacy for severe acne unresponsive to conventional treatments, ISO greatly enhances patients’ quality of life (8).

However, ISO is associated with unique safety concerns compared to other retinoids. Unlike topical retinoids, which have localized effects, ISO is a systemic medication, leading to a broader range of adverse events. Its potent effects on acne treatment are accompanied by significant risks such as teratogenicity, psychiatric side effects, and long-term impacts on lipid metabolism, distinguishing it from other retinoid therapies. Despite its clinical benefits, ISO’s use is constrained by its adverse effects, some of which are severe. Teratogenicity, the most significant concern, affects 20–30% of fetuses exposed in utero, potentially resulting in serious congenital anomalies such as craniofacial defects, cardiovascular malformations, and central nervous system developmental abnormalities (9, 10). Psychiatric effects, including depression and suicidal ideation, have also garnered attention, placing ISO among the top 10 drugs associated with psychiatric adverse events in the FDA database (11). However, conflicting evidence suggests that similar psychiatric symptoms occur in acne patients without ISO treatment, complicating causal interpretation (12). Additionally, milder dose-dependent side effects, such as mucosal dryness, transient telogen effluvium, hypertriglyceridemia, and elevated creatine phosphokinase (CPK) levels, further limit its use (12, 13).

Although ISO has been extensively studied, gaps remain in understanding its safety profile in real-world settings due to limitations like small sample sizes and incomplete adverse event (AE) data. This is where pharmacovigilance plays a crucial role, providing ongoing surveillance of drug safety in larger, more diverse populations, beyond the controlled conditions of clinical trials. Using the FDA Adverse Event Reporting System (FAERS), this study provides the first comprehensive analysis of ISO-associated AEs over two decades. Identifying novel safety signals through pharmacovigilance helps address these knowledge gaps, offering new insights ISO’s real-world safety profile, and supports clinicians in making informed decisions to mitigate risks and improve patient care.

## 2 Materials and methods

### 2.1 Data sources and processing

FAERS database is updated quarterly and consists of seven datasets: patient demographic information (DEMO), drug information (DRUG), indication information (INDI), adverse event information (REAC), reporting source information (RPSR), patient outcomes (OUTC), and treatment start and end dates (THER). These datasets are linked via PRIMARYID (report identifier) to construct complete case records.

**Figure 1:**
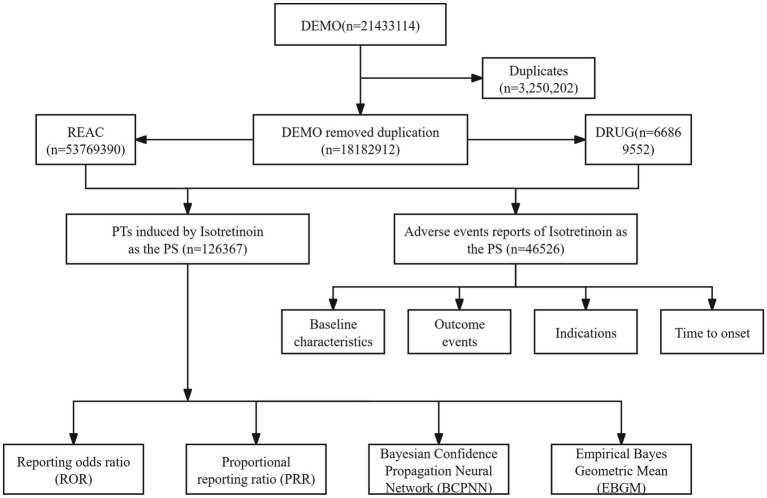
Flow diagram of the study design.

This study collected FAERS data from the first quarter of 2004 to the second quarter of 2024 for pharmacovigilance analysis. Duplicate reports were removed following the FDA-recommended approach, prioritizing the most recent or comprehensive reports based on PRIMARYID, FDA_DT (report date), and CASEID (case identifier), in that order. Given the diversity in reporters and the complexity of drug records, the same drug may appear in the DRUG dataset under different trade or generic names. To comprehensively capture all relevant ISO reports, we searched the DRUG dataset using all known trade and generic names for ISO, including “Absorica,” “Accutane,” “Amnesteem,” “Claravis,” “Clarus,” “Epuris,” “Myorisan,” “Sotret,” “Zenatane,” and “ISO.” Reports listing ISO as a concomitant medication rather than the primary suspect (PS) drug were excluded to minimize potential confounding. The Medical Dictionary for Regulatory Activities (MedDRA) was used to standardize the classification of AEs. MedDRA provides a five-level hierarchy: Lowest Level Terms (LLT), Preferred Terms (PT), High-Level Terms (HLT), High-Level Group Terms (HLGT), and System Organ Classes (SOC). FAERS employs PTs to encode AEs in the REAC dataset. AEs not listed on the ISO drug label were flagged as unlabeled events. Additionally, AEs classified as Important Medical Events (IME) were identified using the MedDRA version 26.1 IME list (Figure 1).

FAERS categorizes symptoms such as cheilitis, cracked lips, and lip inflammation under gastrointestinal disorders rather than skin and mucosal disorders. This categorization was retained to align with FAERS standards, as inflammatory bowel disease (IBD), a potential adverse effect of ISO, may also result in these symptoms. While this classification could introduce minor misclassification bias, it was deemed reasonable for this analysis and maintained to ensure consistency with the FAERS database.

### 2.2 Statistical analysis

#### 2.2.1 Data deduplication and cleaning

The analysis starts in 2004 because FAERS data became available from that time. This ensures the inclusion of both early and recent adverse event reports, which gives a more complete picture of isotretinoin’s safety profile. Additionally, this time period allows for consistent application of the FDA’s data cleaning and deduplication procedures, ensuring the robustness of our findings.

To address the issue of duplicate or withdrawn reports, we strictly followed the FDA’s official data cleaning guidelines. The deduplication process began by selecting the PRIMARYID, CASEID, and FDA_DT fields. We then sorted records by CASEID, FDA_DT, and PRIMARYID, retaining the most recent report when there were duplicates. If both CASEID and FDA_DT were identical, the record with the higher PRIMARYID was kept.

#### 2.2.2 Methods for signal detection and analysis levels

Disproportionality analysis is widely used to detect signals of adverse drug reactions. In this study, we applied four statistical methods using 2 × 2 contingency tables: Reporting Odds Ratio (ROR), Proportional Reporting Ratio (PRR), Bayesian Confidence Propagation Neural Network (BCPNN), and Empirical Bayes Geometric Mean (EBGM). Each method has distinct strengths. ROR is particularly effective in addressing biases caused by small sample sizes, while PRR performs well in cases of incomplete datasets due to its robustness against missing data. BCPNN enhances signal detection accuracy by integrating multiple data sources and employing cross-validation techniques. MGPS (Multi-item Gamma Poisson Shrinker) is well-suited for identifying signals related to rare events. Combining these four methods ensures reliable and robust results, minimizing the limitations of individual approaches (14). Supplementary Table 1 provides the detailed formulas, calculation methods, and specific thresholds used for each of these four methods, further clarifying their operational details.

The analysis was conducted at both the SOC and PT levels. The SOC-level analysis provided a broad perspective on the impact of ISO on specific organ systems, highlighting areas that may require further investigation. PT-level analysis, in contrast, focused on identifying specific AEs, offering a more detailed understanding of the drug’s safety profile. Combining both analysis levels, this approach provided complementary insights into ISO’s potential risks.

#### 2.2.3 Signal significance criteria

The significance of the signals was determined using specific thresholds for each statistical method. For ROR, a lower limit of 95% confidence interval (CI) > 1 and a count of at least 3 reports were considered significant. For PRR, the criteria were PRR ≥ 2, χ2 ≥ 4, and a minimum of 3 reports. In BCPNN, a Interval Confidence 025 (IC025) > 0 was used, and for EBGM, a threshold of EBGM≥2 was applied. These thresholds were chosen to minimize false positives while ensuring the reliability of the results.

#### 2.2.4 Data processing and software

To ensure accuracy and reduce false-positive findings, only PTs that met the criteria for signal detection across all four methods were considered positive signals. Additionally, the time to onset of AEs was calculated by subtracting the ISO start date from the reported AE occurrence date. Reports with missing dates or negative time-to-onset values were excluded from the analysis. All data processing and statistical analyses were performed using R software (version 4.4.1).

## 3 Results

### 3.1 Baseline characteristics

**Figure 2:**
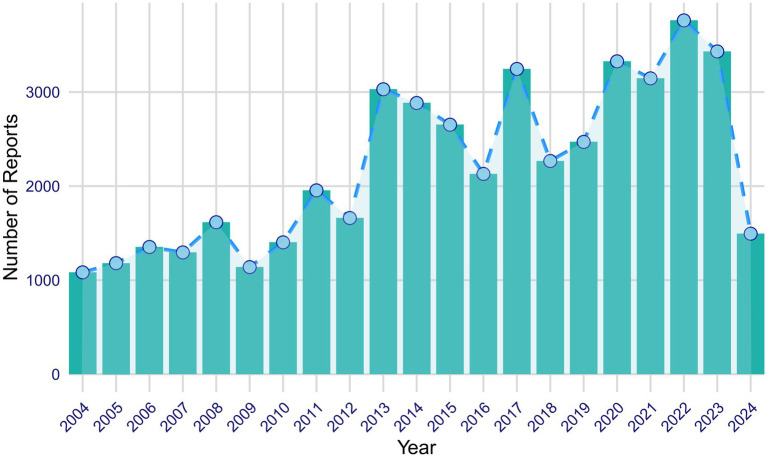
Annual distribution of isotretinoin-related reports from January 2004 to June 2024.

From the first quarter of 2004 to the second quarter of 2024, a total of 46,526 AE reports related to ISO were extracted from the FAERS database. Figure 2 illustrates the annual reporting trends of ISO-related cases over this period. Analysis of the reports revealed that the majority of affected patients were female (55.9%) compared to males (32.9%), while gender was not specified in 11.2% of cases. Most cases involved patients aged 18 to 65 (36.1%), followed by those under 18 (19.0%). Patients over 65 accounted for only 0.5% of the cases, while 44.5% of reports lacked age information. Physicians were the primary reporters of ISO-related AEs (33.2%), followed by consumers (29.7%), other healthcare professionals (12.6%), lawyers (11.1%), general health professionals (6.0%), and pharmacists (2.0%). The role of the reporter was unspecified in 5.3% of cases. Geographically, the majority of reports originated from the United States (81.1%), followed by the United Kingdom (2.2%) and Canada (0.9%). Brazil and Australia accounted for smaller proportions, each contributing 0.5%. The predominance of U.S.-based reports (81.1%) may introduce bias, potentially skewing global trends. This is likely due to stronger reporting infrastructure in the U.S. compared to other regions. Future analyses could assess whether regional differences in healthcare systems or reporting practices affect the results. Regarding clinical outcomes, the most frequently reported outcome was classified as “other serious outcomes” (45.3%). Additionally, 7.4% of cases reported hospitalization; 1.7% recorded death; 1.2% resulted in disability; 1.0% involved life-threatening events; 0.6% led to congenital anomalies; and 0.2% required interventions to prevent permanent damage or impairment. The clinical outcome was not specified in 5.4% of reports. The top five reported indications associated with ISO use were acne (35.0%), cystic acne (5.5%), neuroblastoma (0.4%), rosacea (0.2%), and acne conglobata (0.2%). A detailed summary of the clinical characteristics of ISO-related reports is provided in Table 1.

**Table 1:** Clinical Characteristics of Isotretinoin-Related Reports from January 2004 to June 2024.

Characteristics	Case number	Case Proportion
Number of events	46,526	
Sex		
F	26,008	55.9%
M	15,319	32.9%
Missing	5,199	11.2%
Age(year)		
<18	8,829	19.0%
>85	4	0.0%
18–65	16,789	36.1%
66–85	216	0.5%
Missing	20,688	44.5%
Reported person		
Consumer	13,838	29.7%
Health professional	2,798	6.0%
Lawyer	5,175	11.1%
Physician	15,458	33.2%
Other health-professional	5,855	12.6%
Pharmacist	912	2.0%
Registered Nurse	22	0.0%
Missing	2,468	5.3%
Reported countries (top five)		
United States	37,711	81.1%
United Kingdom	1,026	2.2%
Canada	437	0.9%
Brazil	235	0.5%
Australia	226	0.5%
Outcome		
Death	795	1.7%
Life-Threatening	470	1.0%
Hospitalization	3,435	7.4%
Disability	573	1.2%
Congenital Anomaly	259	0.6%
Required Intervention to Prevent Permanent Impairment/Damage	111	0.2%
Other Serious	21,084	45.3%
Missing	19,799	42.6%
Indications (top five)		
Acne	16,307	35.0%
Acne cystic	2,545	5.5%
Neuroblastoma	180	0.4%
Rosacea	115	0.2%
Acne conglobata	100	0.2%

### 3.2 SOC-level signals

AEs associated with ISO spanned 27 different SOCs. SOCs that met the disproportionality criteria for at least one analysis method included Skin and Subcutaneous Tissue Disorders (*N* = 8,243, ROR: 1.23), Surgical and Medical Procedures (*N* = 2,696, ROR: 1.60), Reproductive System and Breast Disorders (*N* = 1,263, ROR: 1.22), and Eye Disorders (*N* = 3,040, ROR: 1.21). SOCs meeting the criteria for two algorithms were Congenital, Familial, and Genetic Disorders (*N* = 797, ROR: 2.06, PRR: 2.06). SOCs that met the criteria for three algorithms included Psychiatric Disorders (*N* = 21,084, ROR: 3.32, PRR: 2.93, EBGM: 2.92) and Gastrointestinal Disorders (*N* = 27,699, ROR: 3.00, PRR: 2.56, EBGM: 2.55). SOCs meeting the criteria for all four algorithms were Pregnancy, Puerperium, and Perinatal Conditions (*N* = 5,907, ROR: 11.65, PRR: 11.16, EBGM: 10.90, IC: 3.45). Detailed information on all SOC-level signals meeting at least one disproportionality criterion is presented in Table 2.

**Table 2:** Isotretinoin-related signals at the SOC level meeting at least one disproportionality analysis criterion.

SOC	Case numbers	ROR (95%Cl)	PRR (χ^2^)	EBGM (EBGM05)	IC (IC025)
Psychiatric disorders	21,084	**3.32 (3.27–3.37)**	**2.93 (28237.60)**	**2.92 (2.88)**	1.54(−0.12)
Skin and subcutaneous tissue disorders	8,243	**1.23 (1.20–1.26)**	1.21 (329.39)	1.21 (1.19)	0.28(−1.39)
Surgical and medical procedures	2,696	**1.60 (1.54–1.66)**	1.59 (593.04)	1.59 (1.54)	0.67(−1.00)
Gastrointestinal disorders	27,699	**3.00 (2.96–3.04)**	**2.56 (28609.71)**	**2.55 (2.52)**	1.35(−0.32)
Pregnancy, puerperium and perinatal conditions	5,907	**11.65 (11.35–11.97)**	**11.16 (53448.29)**	**10.90 (10.66)**	**3.45 (1.78)**
Reproductive system and breast disorders	1,263	**1.22 (1.15–1.29)**	1.22 (48.53)	1.21 (1.16)	0.28(−1.39)
Congenital, familial and genetic disorders	797	**2.07 (1.93–2.22)**	**2.06 (435.88)**	2.06 (1.94)	1.04(−0.62)
Eye disorders	3,040	**1.21 (1.17–1.25)**	1.20 (107.56)	1.20 (1.17)	0.27(−1.40)

SOC: system organ class; ROR: reporting odds ratios; 95% CI: 95% confidence interval; PRR: proportional reporting ratios; IC: information component; EBGM: empirical Bayesian geometric mean; EBGM05: empirical Bayesian geometric mean lower 95% CI for the posterior distribution. Bolded terms indicate signals meeting the specified algorithm.

### 3.3 PT-level signals

At the PT level, 445 AEs demonstrated significant signals across all four algorithms, spanning 26 SOCs. The five most frequently reported PTs were IBD (N = 4,570), Irritable Bowel Syndrome (*N* = 1,770), Dry Lips (*N* = 1,669), Elevated Blood Triglycerides (N = 1,585), and Gastrointestinal Injury (*N* = 1,150). Of these, IBD, dry lips, and gastrointestinal injury were included in ISO’s drug label. The ten PTs with the strongest signal strengths were IBD (ROR: 492.20), Gastrointestinal Injury (ROR: 383.38), Acne fulminans (ROR: 356.76), Childhood Depression (ROR: 318.38), Nasal Vestibulitis (ROR: 254.76), Ulcerative Proctitis (ROR: 238.80), Premature Epiphyseal Closure (ROR: 206.18), Sicca Syndrome (ROR: 188.08), Epiphyseal Plate Injury (ROR: 141.51), and Cracked Lips (ROR: 140.85). Notably, only three of these ten signals—IBD, gastrointestinal injury, and acne fulminans—were included on the drug label.

Several significant AEs that were not listed on ISO’s label were identified, including Childhood Depression, Nasal Vestibulitis, Ulcerative Proctitis, Premature Epiphyseal Closure, Sicca Syndrome, Epiphyseal Plate Injury, and other rare conditions such as Congenital Middle Ear Abnormalities, Diverticular Hernia, Congenital Cerebellar Agenesis, Follicular Dermatitis, Conglobate Acne, and Hibernation Syndrome. Table 3 provides a detailed list of ISO’s top 50 positive PT signals. Notably, FAERS categorizes certain symptoms, such as Cheilitis, Cracked Lips, and Lip Inflammation, under Gastrointestinal Disorders rather than Skin and Mucosal Disorders. This classification, retained for this analysis, may reflect the multifaceted mechanisms through which ISO induces AEs, such as inflammatory bowel disease.

**Table 3:** Top 50 positive signals of isotretinoin ranked by ROR at the PT level.

PT	SOC	Case numbers	ROR (95%Cl)	PRR (χ^2^)	EBGM (EBGM05)	IC (IC025)
Inflammatory bowel disease^a^	Gastrointestinal disorders	4,570	492.20 (471.69–513.60)	474.43 (1019716.35)	224.57 (216.71)	7.81 (6.14)
Gastrointestinal injury^a^	Injury, poisoning and procedural complications	1,150	383.38 (353.99–415.21)	379.90 (229353.05)	200.95 (187.98)	7.65 (5.98)
Acne fulminans^a^	Skin and subcutaneous tissue disorders	63	356.76 (255.20–498.73)	356.58 (12140.63)	194.25 (146.77)	7.60 (5.92)
Pregnancy test false positive^b^	Investigations	169	319.27 (261.51–389.80)	318.85 (30579.43)	182.51 (154.44)	7.51 (5.84)
Childhood depression^a^*	Psychiatric disorders	3	318.38 (71.26–1422.60)	318.37 (542.36)	182.36 (52.11)	7.51 (5.56)
Nasal vestibulitis^a^*	Infections and infestations	30	254.76 (162.01–400.61)	254.70 (4738.2)	159.56 (109.25)	7.32 (5.62)
Proctitis ulcerative^a^*	Gastrointestinal disorders	182	238.80 (199.13–286.36)	238.45 (27556.47)	153.05 (131.47)	7.26 (5.59)
Abortion induced complete^b^*	Surgical and medical procedures	11	233.50 (111.88–487.32)	233.47 (1642.76)	150.98 (81.58)	7.24 (5.50)
Selective abortion^b^*	Surgical and medical procedures	59	217.89 (159.18–298.24)	217.79 (8414.65)	144.28 (110.95)	7.17 (5.49)
Epiphyses premature fusion^a^*	Musculoskeletal and connective tissue disorders	83	206.18 (158.62–268.00)	206.04 (11401.87)	139.04 (111.65)	7.12 (5.44)
Xerosis^a^*	General disorders and administration site conditions	316	188.08 (164.74–214.73)	187.61 (40677.96)	130.42 (116.73)	7.03 (5.36)
Epiphyseal injury^a^*	Injury, poisoning and procedural complications	7	141.51 (60.15–332.88)	141.50 (732.41)	106.37 (52.00)	6.73 (4.98)
Infant^b^	Social circumstances	6	141.51 (56.17–356.49)	141.50 (627.78)	106.37 (49.10)	6.73 (4.97)
Lip dry^a^	Gastrointestinal disorders	1,669	140.85 (133.24–148.89)	139.00 (172282.62)	104.96 (100.19)	6.71 (5.05)
Anomaly of middle ear congenital^a^*	Congenital, familial and genetic disorders	4	121.29 (39.92–368.49)	121.29 (371.14)	94.56 (37.31)	6.56 (4.77)
Diverticular hernia^a^*	Gastrointestinal disorders	3	115.78 (32.30–415.00)	115.77 (268.2)	91.18 (31.33)	6.51 (4.69)
Anotia^a^	Congenital, familial and genetic disorders	17	109.36 (64.17–186.37)	109.34 (1451.17)	87.15 (55.79)	6.45 (4.75)
Unintended pregnancy^b^	Pregnancy, puerperium and perinatal conditions	1742	100.07 (94.96–105.45)	98.70 (136714.18)	80.27 (76.83)	6.33 (4.66)
Congenital cerebellar agenesis^a^*	Congenital, familial and genetic disorders	8	99.89 (46.24–215.78)	99.88 (633.97)	81.05 (42.55)	6.34 (4.62)
Dermatitis papillaris capillitii^a^*	Skin and subcutaneous tissue disorders	3	97.96 (27.92–343.78)	97.96 (233.93)	79.78 (27.91)	6.32 (4.51)
Acne conglobata^a^*	Skin and subcutaneous tissue disorders	12	96.12 (51.37–179.87)	96.11 (920.96)	78.55 (46.50)	6.30 (4.59)
Hypertrophic anal papilla^a^*	Gastrointestinal disorders	4	94.34 (31.93–278.75)	94.33 (302.22)	77.36 (31.25)	6.27 (4.50)
Abortion induced^b^*	Surgical and medical procedures	1,547	93.38 (88.36–98.68)	92.25 (114720.77)	75.96 (72.53)	6.25 (4.58)
Human chorionic gonadotropin negative^b^*	Investigations	3	84.90 (24.58–293.28)	84.90 (207.28)	70.92 (25.14)	6.15 (4.36)
Drug exposure before pregnancy^b^*	Injury, poisoning and procedural complications	156	82.67 (69.64–98.15)	82.57 (10524.11)	69.29 (60.02)	6.11 (4.45)
Hypersomnia-bulimia syndrome^a^*	Psychiatric disorders	4	80.86 (27.76–235.57)	80.86 (265.00)	68.08 (27.83)	6.09 (4.33)
Delusional disorder, somatic type^a^*	Psychiatric disorders	3	79.60 (23.19–273.17)	79.59 (196.06)	67.18 (23.94)	6.07 (4.28)
Sapho syndrome^a^*	Musculoskeletal and connective tissue disorders	31	77.89 (53.10–114.23)	77.87 (1987.68)	65.95 (47.87)	6.04 (4.37)
Congenital optic nerve anomaly^a^*	Congenital, familial and genetic disorders	4	77.18 (26.60–223.99)	77.18 (254.51)	65.46 (26.84)	6.03 (4.28)
Cheilosis^a^	Gastrointestinal disorders	6	74.92 (31.45–178.44)	74.91 (371.92)	63.82 (30.87)	6.00 (4.27)
Hemihypertrophy^a^*	Congenital, familial and genetic disorders	3	70.75 (20.84–240.20)	70.75 (176.82)	60.79 (21.86)	5.93 (4.15)
Chapped lips^a^	Gastrointestinal disorders	539	69.69 (63.61–76.35)	69.40 (31232.04)	59.79 (55.39)	5.90 (4.24)
Congenital anomaly of inner ear^a^*	Congenital, familial and genetic disorders	3	67.03 (19.83–226.51)	67.03 (168.52)	58.02 (20.95)	5.86 (4.09)
Investigation noncompliance^b^*	Social circumstances	9	64.76 (32.11–130.59)	64.75 (490.16)	56.32 (31.31)	5.82 (4.11)
Intestinal haemorrhage^a^*	Gastrointestinal disorders	629	59.65 (54.87–64.86)	59.36 (31666.51)	52.20 (48.67)	5.71 (4.04)
Epiphyseal disorder^a^*	Musculoskeletal and connective tissue disorders	5	58.96 (23.14–150.25)	58.96 (250.14)	51.89 (23.72)	5.70 (3.97)
Pregnancy^b^	Pregnancy, puerperium and perinatal conditions	2,282	58.29 (55.78–60.92)	57.26 (111182.30)	50.57 (48.74)	5.66 (3.99)
Perirectal abscess^a^*	Infections and infestations	126	57.57 (47.79–69.35)	57.51 (6162.09)	50.77 (43.45)	5.67 (4.00)
Rectal polyp^a^*	Gastrointestinal disorders	121	55.28 (45.74–66.82)	55.23 (5701.42)	48.99 (41.80)	5.61 (3.95)
Cerebellar hypoplasia^a^*	Congenital, familial and genetic disorders	18	54.20 (33.18–88.52)	54.19 (833.39)	48.17 (31.95)	5.59 (3.91)
Ectopic pregnancy termination^b^*	Surgical and medical procedures	6	54.19 (23.17–126.76)	54.19 (277.80)	48.17 (23.66)	5.59 (3.88)
Anal polyp^a^*	Gastrointestinal disorders	11	50.21 (26.88–93.81)	50.21 (474.41)	45.00 (26.68)	5.49 (3.80)
Pregnancy test positive^b^*	Investigations	37	50.04 (35.59–70.35)	50.02 (1590.14)	44.85 (33.73)	5.49 (3.81)
Enterocolonic fistula^a^*	Gastrointestinal disorders	12	49.46 (27.20–89.93)	49.46 (510.27)	44.40 (26.92)	5.47 (3.79)
Vital dye staining cornea present^b^*	Investigations	3	48.98 (14.83–161.83)	48.98 (126.42)	44.02 (16.19)	5.46 (3.71)
Blood triglycerides increased^a^*	Investigations	1,585	47.14 (44.74–49.66)	46.56 (63691.64)	42.05 (40.26)	5.39 (3.73)
Irritable bowel syndrome^a^*	Gastrointestinal disorders	1770	46.61 (44.36–48.96)	45.97 (70275.39)	41.57 (39.89)	5.38 (3.71)
Pouchitis^a^*	Gastrointestinal disorders	71	46.39 (36.31–59.28)	46.37 (2841.37)	41.90 (34.13)	5.39 (3.72)
Cheilitis^a^	Gastrointestinal disorders	477	46.20 (42.03–50.79)	46.03 (18958.85)	41.62 (38.46)	5.38 (3.71)
Sacroiliitis^a^*	Musculoskeletal and connective tissue disorders	168	45.43 (38.74–53.26)	45.37 (6585.68)	41.08 (35.96)	5.36 (3.69)

PT: preferred term; SOC: system organ class; ROR: reporting odds ratios; 95% CI, 95% confidence interval; PRR: proportional reporting ratios; IC, information component; EBGM, empirical Bayesian geometric mean; EBGM05, empirical Bayesian geometric mean lower 95% CI for the posterior distribution. * Adverse events not listed on the drug label.

a Direct adverse events.

b Background events. These refer to events not directly related to the drug’s action but reflecting the patient’s background or management context. They are recorded as supplementary information to provide a comprehensive understanding of the patient’s medication history rather than as drug-induced adverse reactions.

### 3.4 IME signals

Among the 445 PTs identified across all four disproportionality methods, 50 were flagged as IMEs based on the MedDRA version 26.1 IME list (Table 4). Only 5 of these IMEs (10.0%) were included on ISO’s drug label. The identified IME signals were primarily associated with the following SOCs: Congenital, Familial, and Genetic Disorders (*N* = 18, 36.0%), Gastrointestinal Disorders (N = 10, 20.0%), and Pregnancy, Puerperium, and Perinatal Conditions (*N* = 7, 14.0%). Notably, 17 of the IMEs under the Congenital, Familial, and Genetic Disorders SOC were not included on the drug label, highlighting previously unrecognized serious AEs in clinical practice.

**Table 4:** Top 50 IME-related positive signals of isotretinoin ranked by ROR at the PT level.

PT	SOC	Case numbers	ROR (95%Cl)	PRR (χ^2^)	EBGM (EBGM05)	IC (IC025)
Inflammatory bowel disease^a^	Gastrointestinal disorders	4,570	492.20 (471.69–513.60)	474.43 (1019716.35)	224.57 (216.71)	7.81 (6.14)
Acne fulminans^a^	Skin and subcutaneous tissue disorders	63	356.76 (255.2–498.73)	356.58 (12140.63)	194.25 (146.77)	7.60 (5.92)
Proctitis ulcerative^a^*	Gastrointestinal disorders	182	238.80 (199.13–286.36)	238.45 (27556.47)	153.05 (131.47)	7.26 (5.59)
Epiphyses premature fusion^a^*	Musculoskeletal and connective tissue disorders	83	206.18 (158.62–268.00)	206.04 (11401.87)	139.04 (111.65)	7.12 (5.44)
Anomaly of middle ear congenital^a^*	Congenital, familial and genetic disorders	4	121.29 (39.92–368.49)	121.29 (371.14)	94.56 (37.31)	6.56 (4.77)
Anotia^a^	Congenital, familial and genetic disorders	17	109.36 (64.17–186.37)	109.34 (1451.17)	87.15 (55.79)	6.45 (4.75)
Congenital cerebellar agenesis^a^*	Congenital, familial and genetic disorders	8	99.89 (46.24–215.78)	99.88 (633.97)	81.05 (42.55)	6.34 (4.62)
Sapho syndrome^a^*	Musculoskeletal and connective tissue disorders	31	77.89 (53.10–114.23)	77.87 (1987.68)	65.95 (47.87)	6.04 (4.37)
Congenital optic nerve anomaly^a^*	Congenital, familial and genetic disorders	4	77.18 (26.60–223.99)	77.18 (254.51)	65.46 (26.84)	6.03 (4.28)
Hemihypertrophy^a^*	Congenital, familial and genetic disorders	3	70.75 (20.84–240.20)	70.75 (176.82)	60.79 (21.86)	5.93 (4.15)
Congenital anomaly of inner ear^a^*	Congenital, familial and genetic disorders	3	67.03 (19.83–226.51)	67.03 (168.52)	58.02 (20.95)	5.86 (4.09)
Intestinal haemorrhage^a^*	Gastrointestinal disorders	629	59.65 (54.87–64.86)	59.36 (31666.51)	52.20 (48.67)	5.71 (4.04)
Perirectal abscess^a^*	Infections and infestations	126	57.57 (47.79–69.35)	57.51 (6162.09)	50.77 (43.45)	5.67 (4.00)
Cerebellar hypoplasia^a^*	Congenital, familial and genetic disorders	18	54.20 (33.18–88.52)	54.19 (833.39)	48.17 (31.95)	5.59 (3.91)
Enterocolonic fistula^a^*	Gastrointestinal disorders	12	49.46 (27.20–89.93)	49.46 (510.27)	44.40 (26.92)	5.47 (3.79)
Colitis ulcerative^a^*	Gastrointestinal disorders	3,413	43.52 (41.99–45.09)	42.37 (125430.50)	38.61 (37.48)	5.27 (3.61)
Toxic goitre^a^*	Endocrine disorders	3	41.08 (12.56–134.38)	41.08 (106.96)	37.54 (13.93)	5.23 (3.49)
Anophthalmos^a^*	Congenital, familial and genetic disorders	3	39.80 (12.19–129.97)	39.80 (103.74)	36.47 (13.55)	5.19 (3.45)
Device related bacteraemia^a^*	Infections and infestations	17	36.64 (22.32–60.13)	36.63 (542.40)	33.80 (22.33)	5.08 (3.40)
Ear malformation^a^*	Congenital, familial and genetic disorders	36	36.40 (25.89–51.16)	36.39 (1141.08)	33.59 (25.27)	5.07 (3.40)
Traumatic delivery^a^*	Pregnancy, puerperium and perinatal conditions	3	35.38 (10.89–114.88)	35.37 (92.50)	32.73 (12.22)	5.03 (3.31)
Anal stenosis^a^*	Gastrointestinal disorders	35	33.62 (23.83–47.44)	33.61 (1026.28)	31.22 (23.41)	4.96 (3.29)
Interruption of aortic arch^a^*	Congenital, familial and genetic disorders	4	30.87 (11.19–85.19)	30.87 (107.78)	28.85 (12.34)	4.85 (3.14)
Anorectal stenosis^a^*	Gastrointestinal disorders	3	28.30 (8.79–91.07)	28.30 (74.07)	26.59 (10.00)	4.73 (3.02)
Paternal drugs affecting foetus^b^*	Injury, poisoning and procedural complications	22	26.69 (17.35–41.06)	26.68 (511.69)	25.16 (17.55)	4.65 (2.98)
Anomaly of external ear congenital^a^*	Congenital, familial and genetic disorders	16	23.92 (14.45–39.58)	23.92 (332.58)	22.69 (14.89)	4.50 (2.83)
Dandy-walker syndrome^a^*	Congenital, familial and genetic disorders	13	22.90 (13.11–40.02)	22.90 (258.31)	21.78 (13.65)	4.44 (2.77)
Teratogenicity^a^*	Congenital, familial and genetic disorders	14	22.77 (13.30–38.99)	22.77 (276.56)	21.66 (13.81)	4.44 (2.76)
Choroid melanoma^a^*	Neoplasms benign, malignant and unspecified (incl cysts and polyps)	5	21.66 (8.82–53.20)	21.66 (93.74)	20.66 (9.74)	4.37 (2.68)
Keratoconus^a^*	Eye disorders	14	21.08 (12.32–36.05)	21.07 (255.05)	20.12 (12.84)	4.33 (2.66)
Colonic fistula^a^*	Gastrointestinal disorders	24	20.59 (13.67–31.01)	20.58 (426.46)	19.68 (13.97)	4.30 (2.63)
Crohn’s disease^a^*	Gastrointestinal disorders	2,529	19.90 (19.11–20.71)	19.52 (42527.07)	18.70 (18.09)	4.23 (2.56)
Cholangitis sclerosing^a^*	Hepatobiliary disorders	73	18.35 (14.51–23.19)	18.34 (1147.03)	17.62 (14.48)	4.14 (2.47)
Pregnancy on oral contraceptive^b^*	Pregnancy, puerperium and perinatal conditions	93	18.00 (14.62–22.15)	17.99 (1431.26)	17.30 (14.54)	4.11 (2.45)
Conjoined twins^a^*	Congenital, familial and genetic disorders	4	17.87 (6.57–48.61)	17.87 (61.14)	17.19 (7.44)	4.10 (2.41)
Congenital eye disorder^a^*	Congenital, familial and genetic disorders	9	16.83 (8.65–32.77)	16.83 (128.90)	16.23 (9.29)	4.02 (2.34)
Pregnancy with injectable contraceptive^b^*	Pregnancy, puerperium and perinatal conditions	8	16.57 (8.17–33.57)	16.57 (112.62)	15.98 (8.85)	4.00 (2.32)
Growth failure^a^*	Musculoskeletal and connective tissue disorders	11	16.39 (8.97–29.92)	16.38 (152.99)	15.81 (9.55)	3.98 (2.31)
Shoulder dystocia^a^*	Pregnancy, puerperium and perinatal conditions	5	16.33 (6.68–39.89)	16.33 (69.28)	15.76 (7.46)	3.98 (2.29)
Pregnancy on contraceptive^b^*	Pregnancy, puerperium and perinatal conditions	50	16.07 (12.12–21.32)	16.07 (680.73)	15.52 (12.25)	3.96 (2.29)
Foetal heart rate increased^a^*	Investigations	3	15.53 (4.91–49.15)	15.53 (39.35)	15.02 (5.73)	3.91 (2.21)
Ectopic pregnancy^a^*	Pregnancy, puerperium and perinatal conditions	82	12.99 (10.43–16.19)	12.98 (880.10)	12.63 (10.51)	3.66 (1.99)
Abortion^a^	Pregnancy, puerperium and perinatal conditions	79	12.79 (10.22–16.00)	12.78 (832.79)	12.44 (10.31)	3.64 (1.97)
Congenital neurological disorder^a^*	Congenital, familial and genetic disorders	3	11.79 (3.74–37.14)	11.79 (28.83)	11.50 (4.40)	3.52 (1.84)
Suicidal ideation^a^	Psychiatric disorders	2,175	11.78 (11.29–12.30)	11.60 (20534.37)	11.32 (10.92)	3.50 (1.83)
Anal abscess^a^*	Infections and infestations	126	11.53 (9.66–13.76)	11.52 (1178.28)	11.24 (9.69)	3.49 (1.82)
Gastrointestinal oedema^a^*	Gastrointestinal disorders	40	11.10 (8.11–15.20)	11.10 (358.17)	10.84 (8.34)	3.44 (1.77)
Choanal atresia^a^*	Congenital, familial and genetic disorders	3	10.98 (3.49–34.54)	10.98 (26.52)	10.73 (4.11)	3.42 (1.74)
Rectal abscess^a^*	Infections and infestations	40	10.94 (7.99–14.97)	10.93 (351.95)	10.68 (8.22)	3.42 (1.75)
Congenital central nervous system anomaly^a^*	Congenital, familial and genetic disorders	24	10.78 (7.19–16.17)	10.78 (207.70)	10.54 (7.51)	3.40 (1.73)

PT: preferred term; SOC: system organ class; ROR: reporting odds ratios; 95% CI, 95% confidence interval; PRR: proportional reporting ratios; IC, information component; EBGM, empirical Bayesian geometric mean; EBGM05, empirical Bayesian geometric mean lower 95% CI for the posterior distribution. * Adverse events not listed on the drug label.

a Direct adverse events.

b Background events. These refer to events not directly related to the drug’s action but reflecting the patient’s background or management context. They are recorded as supplementary information to provide a comprehensive understanding of the patient’s medication history rather than as drug-induced adverse reactions.

### 3.5 Time to AE onset

**Figure 3:**
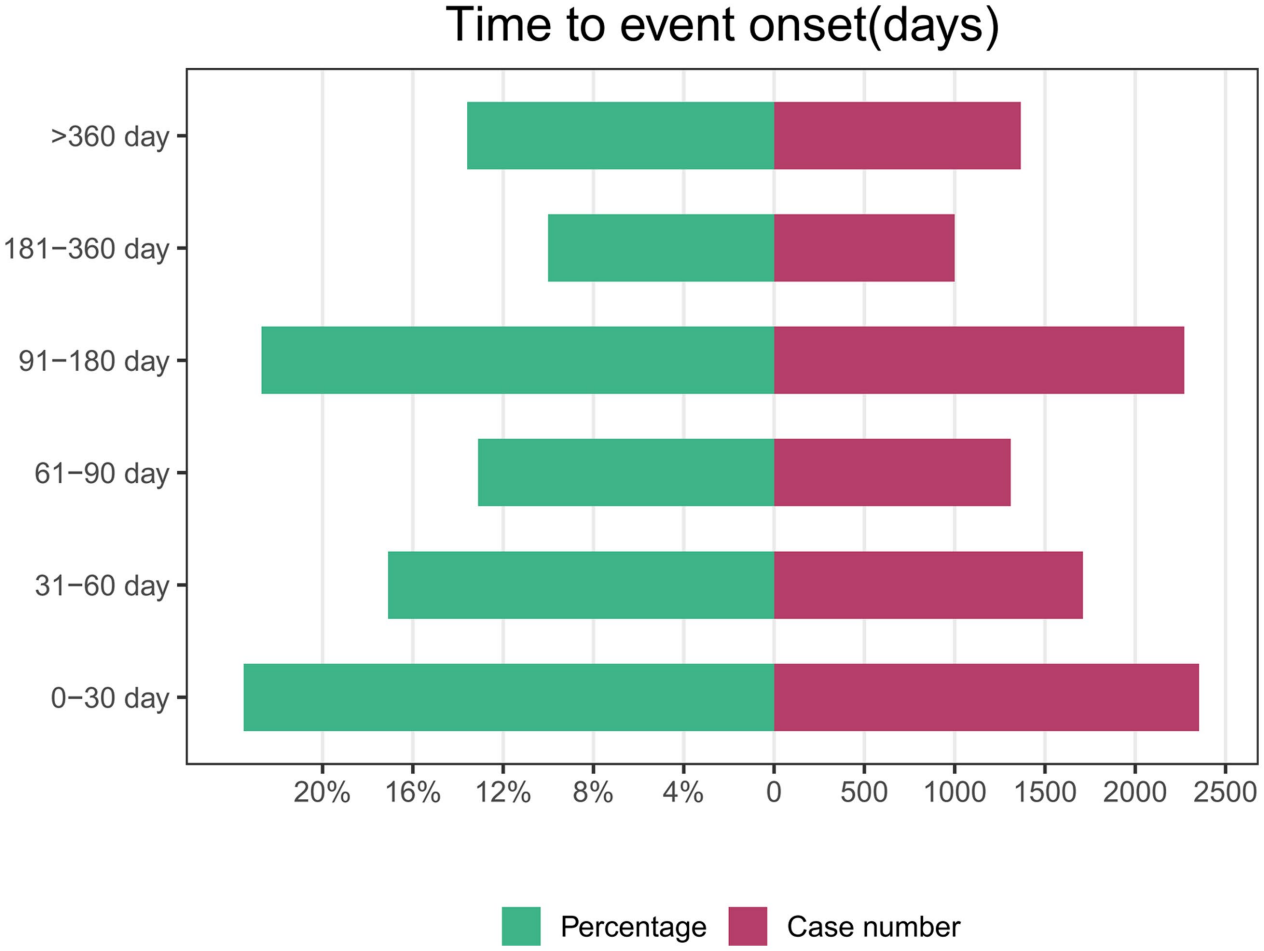
Timing of adverse events associated with isotretinoin.

Of the 46,526 AE reports related to ISO, 10,012 contained complete and accurate time-to-onset information. The median time to AE onset was 81 days, with an interquartile range of 31 to 169 days. Approximately 23.5% of AEs (*N* = 2,354) occurred within the first month of ISO treatment. Interestingly, the incidence of AEs during months 4 to 6 (*N* = 2,272, 22.7%) was comparable to the first month. In contrast, the likelihood of AEs was lowest during months 7 to 12. This pattern suggests that close monitoring is particularly important during the early and mid-treatment phases. The higher AE incidence in these periods may reflect the body’s initial response or dosage adjustments. These findings emphasize the need for more frequent follow-up visits in the first 6 months to detect AEs early and enable timely intervention. Figure 3 illustrates the time-to-onset distribution for ISO-related AEs.

## 4 Discussion

ISO is primarily used to treat severe recalcitrant acne. Its mechanism of action includes inhibiting sebaceous gland lipid synthesis, reducing the proliferation of *Propionibacterium acnes*, exerting anti-inflammatory and immunomodulatory effects, and improving follicular and sebaceous duct keratinization, effectively controlling acne symptoms (15). Beyond its labeled indication for severe acne, evidence suggests that ISO is frequently used off-label to treat mild-to-moderate acne, inflammatory skin disorders, genetic skin conditions, skin cancers, and other dermatologic conditions (2, 13, 16). These observations align with the primary indications reported in this study, which include acne, cystic acne, neuroblastoma, rosacea, and acne conglobata. This versatility may be attributed to ISO’s anti-inflammatory and immunomodulatory properties, such as reducing TLR-2 expression on monocytes, suppressing inflammatory cytokine responses, and exhibiting antitumor characteristics (17, 18).

Our study observed an overall upward trend in the number of AE reports related to ISO from 2004 to 2023, with reports rising from approximately 1,000 in 2004 to over 3,000 in 2023. However, fluctuations were observed throughout this period, with notable declines in 2016 and 2018, which could be attributed to factors such as changes in reporting practices or regulatory adjustments. The highest number of reports was recorded in 2022, likely reflecting increased clinical awareness, changes in reporting practices, or demographic shifts. Additionally, the growing off-label use of ISO, alongside a larger patient population, likely contributed to both the increased volume and the broader spectrum of AE reports. ISO is more commonly used in high-income countries (19), corresponding with the primary reporting regions identified in this study—namely, the United States, the United Kingdom, and Canada. Female patients accounted for 55.9% of reports, a discrepancy likely driven by the widespread use of ISO among women of childbearing age and heightened awareness of its teratogenic risks. These findings underscore the importance of targeted safety measures for women, as ISO’s risks extend beyond congenital anomalies to potential long-term neuropsychological effects in exposed offspring (9).

Disproportionality analysis revealed key signals requiring close attention. SOCs such as “Pregnancy, Puerperium, and Perinatal Conditions” and “Gastrointestinal Disorders” are explicitly flagged in ISO’s drug label, underscoring their clinical relevance. Teratogenicity, a hallmark safety concern of ISO, is largely attributed to its active metabolite, 4-oxo-ISO, which exhibits high toxicity to neural crest cells (20). The teratogenic effects of ISO are further mediated by its isomerization to all-trans-retinoic acid (ATRA), which increases placental transfer and fetal exposure, contributing to its teratogenic potency. Species differences in ISO metabolism play a key role in the variability of teratogenic outcomes. In sensitive species, such as primates, ISO is primarily metabolized into the active 13-cis-4-oxo-retinoic acid, with extensive placental transfer. In contrast, insensitive species like rodents rapidly metabolize ISO, primarily through *β*-glucuronidation, limiting placental transfer and reducing teratogenic risks (21). Furthermore, the teratogenic action of ISO is associated with retinoic acid receptor-mediated gene expression, including Hox gene regulation, retinal metabolism, and the direct nuclear access of 13-cis-retinoic acid, which may further enhance the risk of developmental defects (5, 22, 23). As a result, exposure to ISO during pregnancy can lead to severe congenital anomalies, collectively referred to as “Accutane embryopathy,” encompassing central nervous system defects, craniofacial abnormalities, cardiovascular malformations, thymic aplasia, and other malformations such as limb shortening (9, 10). These findings are consistent with historical observations in animal studies, where ISO was first identified as a teratogen in 1954 (24). In contrast, our study identified several unlabeled IMEs, which have been rarely reported in the literature. These findings reveal additional teratogenic risks that have not been adequately recognized in the context of ISO. In the “Congenital, Familial, and Genetic Disorders,” index, 18 PTs met all four disproportionate criteria. Of these, 94.4% (17 PTs) were unlabeled IMEs, including severe conditions such as interrupted aortic arch, cerebellar hypoplasia, anotia, and Dandy-Walker syndrome. Notably, while anotia and Dandy-Walker syndrome have been rarely reported in earlier studies, they have not been included in ISO’s official list of labeled adverse effects. The discovery of these unlabeled IMEs underscores significant gaps in the clinical recognition of ISO’s teratogenic risks and reveals potential diagnostic blind spots. These findings emphasize the need for improved post-marketing surveillance and updated clinical guidelines to better address these risks. The prolonged half-life of ISO (12.9 h) and its active metabolite (60.75 h), along with enterohepatic circulation, significantly extends fetal exposure even after discontinuation of therapy (25, 26). This pharmacokinetic property further complicates reproductive safety management and underscores the importance of strict adherence to guidelines for contraception and drug discontinuation prior to conception.

In addition to teratogenic risks, our study identified “Gastrointestinal Disorders” as the most frequently reported SOC, including AEs such as IBD, ulcerative proctitis, dry lips, intestinal bleeding, and anal polyps. Evidence suggests that ISO may trigger IBD through mechanisms like mucosal barrier disruption, altered gut microbiota, and immune dysregulation mediated by cytokines such as IL-17 and TNF-*α* (27, 28). While the link between ISO and IBD remains debated, with some attributing IBD to underlying patient susceptibility, growing data support a potential association that warrants further investigation (29, 30). ISO may induce IBD through multiple mechanisms. It can disrupt intestinal immune balance by inhibiting epithelial cell growth and T cell activation (31). Additionally, ISO-activated T cells express α4β7 and CCR9 receptors, which are crucial in gastrointestinal inflammation. ISO also affects neutrophil chemotaxis and reactive oxygen species production, exacerbating intestinal inflammation (28). Furthermore, ISO’s impact on lipid profiles, particularly HDL, may promote monocyte activation and further amplify inflammatory responses (32). Although ISO may promote IBD by damaging the intestinal barrier, retinoic acid (a form of ISO) has been shown to enhance the expression of tight junction proteins, such as occludin, claudin-1, claudin-4, and zonula occludens-1, thereby improving intestinal barrier function and potentially mitigating IBD progression (33). Therefore, ISO’s role in IBD is complex, with the potential to both exacerbate inflammation and, under certain conditions, protect the intestinal barrier (34). Therefore, while the role of ISO in IBD remains unclear, its dual potential to exacerbate or protect the intestinal barrier highlights the need for further research to clarify the mechanisms involved. The FAERS classification of symptoms such as cheilitis and cracked lips under “Gastrointestinal Disorders” illustrates the diverse pathways through which ISO induces AEs. These symptoms may result from local mucosal effects or immune-driven gastrointestinal conditions like IBD. This classification reflects the systemic reach of ISO’s effects, emphasizing the importance of monitoring its impact beyond its traditional focus on skin and mucosal tissues.

Building on the complexity of ISO’s adverse effects, our study identified previously unlabeled psychiatric AEs, including childhood depression, hypersomnolence-bulimia syndrome, and somatic delusional disorder. IME-related psychiatric AEs, such as suicidal ideation, were also observed. These findings highlight the importance of understanding ISO’s potential psychiatric risks, particularly in patients with a history of psychiatric disorders or those predisposed to mood changes. The relationship between ISO and psychiatric disorders remains highly debated. Some studies suggest a connection to depression, anxiety, and suicidal behavior—prompting the FDA to issue a black box warning in 2005 (35). Challenge-dechallenge-rechallenge data indicate a potential causal link between ISO and mood disturbances in certain cases (35–37). The proposed mechanisms include alterations in mood-regulating neurotransmitters, such as serotonin, dopamine, and norepinephrine, triggered by ISO (3, 38). However, findings from a large-scale meta-analysis report no significant increase in the risk of depression or suicide among ISO users (39). Additionally, some studies propose that, by improving acne severity and self-image, ISO may even alleviate mood symptoms in certain individuals (37, 40). In light of these contrasting views, clinicians should remain vigilant for potential mood changes, particularly in high-risk patients with a history of psychiatric conditions. Nevertheless, at the population level, ISO appears to present minimal risk for psychiatric disorders. The contrast between our findings and previous studies highlights the complexity of ISO’s psychiatric effects and reinforces the need for personalized treatment and careful patient management.

AEs associated with ISO therapy occur most frequently during the early (0–30 days, 23.5%) and mid-treatment periods (91–180 days, 22.7%), reflecting distinct patterns in the treatment timeline. Early AEs, such as dry skin and cheilitis, may result from ISO’s rapid suppression of sebaceous gland activity and associated skin dryness. Mid-treatment AEs might reflect cumulative drug exposure and its broader systemic effects. Literature indicates that approximately 85% of patients achieve acne clearance by the fourth month of therapy (41), aligning with the recommended 4–6 month treatment duration in European guidelines (42). The reduction in AEs during later stages may correspond to treatment discontinuation and declining systemic drug concentrations, though this trend requires further investigation. These findings highlight the need for active monitoring and management during the early and mid-treatment phases to optimize patient outcomes.

In light of these findings, we recommend that clinicians implement enhanced pre-treatment counseling, particularly for women of childbearing age, emphasizing the teratogenic risks of ISO and the importance of effective contraception. Furthermore, clinicians should closely monitor patients during the early and mid-treatment phases, when AEs are most frequent. Early AEs, such as dry skin and cheilitis, may require proactive management, while mid-treatment AEs should prompt careful assessment of cumulative drug exposure and systemic effects. Regular follow-ups and close attention to adverse effects will help optimize treatment outcomes and minimize risks.

This study represents the first pharmacovigilance analysis of ISO using the FAERS database, offering a novel real-world perspective on its safety profile. However, the FAERS database, as a spontaneous reporting system, has several inherent limitations. Underreporting is a common issue, as not all adverse events are reported, particularly those that are less severe or do not require medical attention. Additionally, reporting bias may arise as healthcare professionals are more likely to report serious or uncommon adverse events, while less severe or common events may be underreported (43). The classification system in the FAERS database may also affect signal detection at the SOC level, as evidenced by the categorization of symptoms like cheilitis under “Gastrointestinal Disorders.” Furthermore, the lack of causality confirmation in the FAERS database limits our ability to establish definitive cause-and-effect relationships between ISO and adverse events. AEs listed on drug labels are primarily derived from randomized controlled trials (RCTs), which, due to their strict inclusion criteria, may underestimate real-world safety risks (44). Despite these limitations, our study highlights the value of real-world data in uncovering safety signals that might be overlooked in controlled trials, emphasizing the importance of integrating complementary methodologies to refine ISO’s safety evaluation. Finally, to improve the clarity and consistency of adverse event documentation, clinicians should provide detailed descriptions of the event’s onset, duration, severity, and outcome, along with relevant clinical context such as co-medications and patient demographics. Standardizing reporting practices through structured forms and coding systems could also enhance report quality, ultimately improving medication safety monitoring.

## 5 Conclusion

Our study is the first to conduct a pharmacovigilance analysis of ISO using the FAERS database, addressing a critical gap in real-world safety data. We identified several previously unlabeled AEs, including life-threatening IMEs such as Dandy-Walker syndrome and suicidal ideation, emphasizing the need for updated drug labels and improved clinical awareness. The predominance of “Gastrointestinal Disorders” as the most reported SOC further highlights ISO’s systemic impact beyond dermatologic use. This study underscores the importance of real-world pharmacovigilance in identifying overlooked safety signals. It lays the groundwork for future research, including mechanistic studies to validate the findings and understand the pathophysiology of the identified AEs, as well as long-term follow-up to refine ISO’s safety profile. Additionally, the findings highlight the need for a global pharmacovigilance framework to more effectively monitor isotretinoin and similar high-risk drugs. Such a framework would ensure consistent data collection, improve safety signal detection, and facilitate global collaboration to enhance patient safety across diverse healthcare settings.

## Data Availability

The original contributions presented in the study are included in the article/Supplementary material, further inquiries can be directed to the corresponding author.

## Supplementary material

SUPPLEMENTARY TABLE 1 Integrated 2×2 table, formulas, and thresholds for four analytical methods.
